# Advance Directive Completion and Hospital Out-of-Pocket Expenditures

**DOI:** 10.1093/geroni/igab046.2185

**Published:** 2021-12-17

**Authors:** Yujun Zhu, Susan Enguidanos

**Affiliations:** 1 University of Southern California, South Pasadena, California, United States; 2 University of Southern California, Los Angeles, California, United States

## Abstract

Healthcare costs remain high at end of life. Although advance directives (AD) have been shown to improve care congruence with patients’ preferences and lower cost of healthcare services, little is known about the relationship between AD completion and hospital out-of-pocket costs. This study examined whether AD completion was associated with lower hospital out-of-pocket spending at end of life. We used the Health and Retirement Study participants who died between 2000 and 2014 (N=9,228) to examine the association through the use of a two-part analytic model that has been widely used in health economics. We controlled for socioeconomic status, death-related characteristics, and health insurance coverage and imputed missing data using multiple imputation by chained equations. Of the 43.9% of decedents who completed an AD, 90.7% chose to limit care or to be kept comfortable; 78.8% indicated that they wanted to withhold treatment, and 5.6% wanted to prolong life. Having an AD was significantly associated with $632 (95% CI: [-$1,116.47, -$146.71]) lower hospital out-of-pocket costs, with greater savings among younger decedents, dropping from $1,560 (95% CI: [-$2,652, -$268]) at age 50 to $230 (95% CI: [-$445, -$14]) at age 110. Decedents who completed an AD 12 months or less before death had higher out-of-pocket spending ($1,591 on average) than those who completed more than a year before death ($1,001 on average). Our findings have policy implications for physician-patient communication about costs of care and may provide an opportunity for physicians to involve cost-sharing discussions when completing ADs with patients.

